# Visceral leishmaniasis complicating idiopathic CD4^+^ T-cell lymphocytopenia: 2 case reports

**DOI:** 10.1371/journal.pntd.0005412

**Published:** 2017-05-11

**Authors:** Andrew Fox-Lewis, Diana N. J. Lockwood

**Affiliations:** 1 Hospital for Tropical Diseases, University College London Hospitals National Health Service (NHS) Foundation Trust, London, United Kingdom; 2 Clinical Research Department, London School of Hygiene and Tropical Medicine, London, United Kingdom; Ohio State University, UNITED STATES

## Introduction

HIV infection leads to worse treatment outcomes in visceral leishmaniasis (VL) and increases the risk of post-treatment relapse, suggesting that similar effects may be observed in other low CD4^+^ T cell states [1]. Idiopathic CD4^+^ T cell lymphocytopenia (ICL) was first described in 1992 in the setting of the emerging HIV pandemic [2]. It is a rare condition defined as a CD4^+^ T cell count persistently <300 cells/μl in the absence of known secondary causes of lymphopenia, such as HIV infection [3]. VL infection in individuals with ICL is a little-reported clinical entity [4, 5, 6] and here we report in detail our experiences of 2 such patients from the Hospital for Tropical Diseases (HTD). The cases discussed here add an important clinical perspective to the immunological models. One patient has had complex refractory disease managed over the last 12 years, developing both ocular and dermal leishmaniasis and demonstrating how CD4 deficiency without HIV infection affects the clinical course of VL.

### Presentation of Case 1

A 53-year-old British male with known psoriasis and chronic kidney disease (CKD) presented to his general practitioner (GP) in August 2004 with fatigue, night sweats, weight loss, and exertional breathlessness. He lived in the United Kingdom and his lifetime travel history to *Leishmania*-endemic areas consisted of Spain in 2002, Cyprus annually from 1998–2003, and, in June 2004 (2 months prior to presentation), a 2-week holiday to the Greek island of Zakynthos, during which he reported insect bites. He was investigated and found to have pancytopenia, and in October 2004, *Leishmania* amastigotes were identified on a bone marrow biopsy, giving the patient a diagnosis of VL. Polymerase chain reaction (PCR) testing detected *Leishmania donovani* DNA. In November 2004, the patient had his first course of treatment with intravenous (IV) AmBisome (liposomal amphotericin B), leading to a resolution of his symptoms.

In February 2005, the patient relapsed for the first time with recurrence of his presenting symptoms and was treated empirically with a further course of IV AmBisome. Following his second relapse in June 2005, the patient was referred to the HTD for management. Relapse was confirmed with a bone marrow biopsy showing *Leishmania* amastigotes, and in July 2005, the patient had his third treatment course, consisting of oral miltefosine and IV AmBisome.

Between August 2005 and June 2008, the patient was managed with prophylactic IV pentamidine isethionate infusions once every 2 weeks and had a relapse-free period. In January 2006, the patient developed bilateral anterior uveitis and intraocular hypertension of unknown etiology. This was managed with systemic immunosuppression, intraocular steroid injections, and, in 2008, bilateral ocular surgical drainage.

In June 2008, the patient presented with 6 months of new, small, tender, raised erythematous skin lesions over his abdomen and legs, distinct from the non-tender psoriatic patches on his elbows. Numerous *Leishmania* amastigotes were identified on skin biopsy of a lesion, giving the patient a diagnosis of Post-kala-azar dermal leishmaniasis (PKDL) and representing his third clinical relapse. In July 2008, *Leishmania* organisms or DNA were not identified on microscopy and PCR of a splenic aspirate, showing that the patient had not had a systemic recurrence of VL. The patient was not treated at this time.

The patient's vision continued to deteriorate despite aggressive treatment of his anterior uveitis and intraocular hypertension, and he was registered as blind in April 2009. In May 2009, the patient underwent an ocular vitreous aspirate to determine if his visual deterioration could be related to his leishmaniasis. *Leishmania* species DNA was identified on PCR of the aspirate, confirming intraocular leishmaniasis and his fourth clinical relapse. In July 2009, the patient was admitted for treatment with 28 days of IV sodium stibogluconate. After 22 days, the course was halted due to drug toxicity. A repeat biopsy of a skin lesion at this time did not identify *Leishmania* on microscopy or PCR, and so stibogluconate treatment was not continued. *Leishmania* DNA was not identified on PCR of a further ocular vitreous aspirate in October 2009.

Between October 2009 and May 2010, the patient remained off prophylactic pentamidine, which was stopped prior to admission and not restarted following discharge. In May 2010, the patient had a fifth clinical relapse. *Leishmania* amastigotes were visualized on microscopy of a bone marrow biopsy at this time, and *Leishmania donovani* complex DNA was identified on PCR of the sample. The patient was treated with a further course of IV AmBisome in June 2010. A splenic aspirate in July 2010 found no *Leishmania* amastigotes on microscopy, but *Leishmania* DNA continued to be isolated on PCR of the sample. At this time, the patient was re-commenced on prophylactic pentamidine infusions administered once every 3 weeks. In March 2011, *Leishmania* DNA was once again isolated from PCR of an ocular vitreous aspirate, but a splenic aspirate at this time did not identify *Leishmania* organisms or DNA, indicating that the pentamidine infusions were controlling his systemic infection but not the ocular disease.

In August 2011, the patient presented to clinic with new erythematous papular lesions on his abdomen. Skin slit smear microscopy of these revealed amastigotes, confirming further dermal leishmaniasis and his sixth relapse. *Leishmania* DNA was identified on PCR of a splenic aspirate in April 2012, and the patient was managed with a further course of oral miltefosine and IV AmBisome.

During this time, a cause was sought for the patient’s treatment refractory disease. HIV serology was negative and immunoglobulins were within normal range. Lymphocyte subset analysis, consisting of total lymphocyte count, CD3^+^ lymphocyte count (T cell count), CD3^+^CD4^+^ lymphocyte count (helper or CD4^+^ T cell count), and CD3^+^CD8^+^ lymphocyte count (cytotoxic or CD8^+^ T cell count), was undertaken on multiple occasions during the course of the patient’s illness and revealed a persistently low CD4^+^ T cell count of between 40–160 cells/μl along with a low CD8^+^ T cell count of 180–220 cells/μl. Investigations for secondary causes of his persistent lymphopenia were non-diagnostic. There was no history of malnutrition, alcohol excess, radiotherapy, recent surgery, inflammatory bowel disease, or immunosuppressive medication other than intermittent systemic and local corticosteroid treatment for his anterior uveitis and intraocular hypertension. Multiple blood films, bone marrow biopsies, and splenic aspirates found no evidence of hematological malignancy, and cross-sectional imaging did not demonstrate any solid organ malignancies. Screening for autoimmune diseases and sarcoidosis was negative, and cardiac and renal function were grossly normal. When no known secondary cause of lymphopenia could be identified after extensive investigation, the patient was given a diagnosis of ICL.

In August 2012, a post-treatment skin biopsy of a PKDL skin lesion showed mild chronic inflammation only, with no evidence of *Leishmania* on microscopy or PCR, suggesting the patient had cleared the *Leishmania* from his skin. Since this time, his leishmaniasis has been kept in remission by ongoing three-weekly pentamidine prophylaxis, and he has remained relapse-free.

### Presentation of Case 2

A 42-year-old Serbian male with type 2 diabetes mellitus presented on July 2014 with fevers, drenching night sweats, weight loss, and fatigue. The patient was born in Belgrade, Serbia, and had been living in the UK since 2011. Lifetime travel history to *Leishmania*-endemic regions included holidays to Gibraltar in 2008, Spain in 2011, and Greece in 2013.

Widespread lymphadenopathy and hepatosplenomegaly was found on cross-sectional imaging, and the patient was referred to the HTD with a suspected diagnosis of VL. *Leishmania* serology by direct agglutination test (DAT) was strongly positive at a titre of 1:102,400 (a titre exceeding 1:1,600 is considered positive). *Leishmania* amastigotes were subsequently identified on a lymph node biopsy, confirming the diagnosis of VL, although PCR was unable to confirm the species. The patient was treated with a course of IV AmBisome.

Due to our experiences with the patient from Case 1, HIV testing and lymphocyte subsets are now requested as baseline investigations for all patients with VL managed at the HTD, and the patient from Case 2 underwent these tests accordingly. HIV serology was negative, but lymphocyte subset analysis revealed a persistently low CD4^+^ T cell count of 90–160 cells/μl, with normal CD8^+^ T cell levels. As with the patient from Case 1, no secondary causes of his persistent lymphopenia could be identified, giving the patient a diagnosis of ICL.

The patient had a good clinical response to a single course of treatment with IV AmBisome and remains in remission since 2014, without prophylaxis. We continue to monitor him for relapses.

### Case discussion

*Leishmania* infection is controlled in the host by a T cell–mediated immune response, with CD4^+^, CD8^+^, and natural killer T cells contributing to the immunological processes involved [7]. CD4^+^ T cells are thought to play a major role, with animal models of VL suggesting that CD4^+^ T cells are responsible for organ-specific antiparasitic immunity, immune surveillance, and parasite suppression [8, 9]. Additionally, certain cytokines have emerged as having key pathogenic or protective roles in VL. Interleukin 10 (IL-10), produced by dendritic cells (DCs) and regulatory T cells (Tr1), acts to suppress antiparasitic immune mechanisms in human VL, with IL-10 deficiency protecting against infection and blockade of IL-10 signaling, enhancing antiparasitic immunity [7, 8]. Conversely, interferon gamma (IFNγ) appears crucial for host control of the disease, with elevated levels correlating with recovery, and neutralization of IFNγ in vitro promoting parasite growth [7, 10]. The main source of IFNγ production in the context of VL is CD4^+^ Th1 cells, which are in turn induced by DC interleukin 12 (IL-12) production [8, 10]. Deficiency of CD4^+^ T cells would thus be expected to result in reduced IFNγ production and impaired ability of the host to control infection.

The cases discussed here add a clinical perspective to the immunological models, suggesting that patients with ICL are predisposed to developing chronic VL following exposure and have reduced ability to clear *Leishmania* parasites, leading to relapse despite treatment. The patient from Case 2 responded successfully to a single course of treatment. In contrast, the patient from Case 1, who had a lower nadir CD4^+^ T cell count, a low CD8^+^ T cell count, and received concurrent steroid therapy, had complex treatment refractory disease with multiple relapses, including ocular and dermal disease, and is held in remission by long-term pentamidine prophylaxis. This suggests that these factors may have had a cumulative deleterious effect on the clinical course of his disease.

It has been our experience at the HTD that immunodeficiency is a risk factor for the development of VL, with two-thirds of the VL patients we see having some form of immunodeficiency identified [11]. Given that AmBisome treatment has a 95% cure rate [12], patients who relapse following treatment should be investigated for underlying immunodeficiency. We suggest that, where available, both HIV testing and lymphocyte subset analysis should be considered as baseline investigations for all patients diagnosed with VL—adopting this strategy, we have thus far identified 2 patients with underlying ICL. Additionally, in patients who relapse following treatment, in whom baseline HIV testing is negative and lymphocyte subset analysis is normal, these tests should be repeated and other causes of immunodeficiency sought and corrected where possible, such as immunosuppressive medications, environmental toxins, malnutrition, alcohol excess, malignancy, organ failure, protein-losing disorders, and metabolic and endocrine disorders.

Patients with VL and any form of immunodeficiency, such as ICL or immunosuppressive therapy, must be monitored closely for signs of relapse and may benefit from secondary prophylaxis, which has been found to be effective in preventing relapse in immunocompromised patients [4]. IFNγ therapy has had some success in treatment of other infections in the context of ICL and so may have a future therapeutic role in the management of VL infection in patients with ICL [13, 14].

**Key learning points**

- Idiopathic CD4^+^ T cell lymphocytopenia is a rare condition characterized by persistently low CD4^+^ T cell count in the absence of known secondary causes of lymphopenia.
- Immunodeficiency is a risk factor for the development of visceral leishmaniasis; where available, HIV serology and lymphocyte subset analysis should be performed as baseline investigations for all patients diagnosed with visceral leishmaniasis.
- In patients who relapse following treatment, in whom baseline HIV testing is negative and lymphocyte subset analysis is normal, these tests should be repeated and other causes of immunodeficiency sought and corrected where possible.
- Patients with visceral leishmaniasis and any form of immunodeficiency, such as idiopathic CD4^+^ T cell lymphocytopenia or immunosuppressive therapy, must be monitored closely for signs of relapse and may benefit from secondary prophylaxis.
